# Bridging scales through multiscale modeling: a case study on protein kinase A

**DOI:** 10.3389/fphys.2015.00250

**Published:** 2015-09-09

**Authors:** Britton W. Boras, Sophia P. Hirakis, Lane W. Votapka, Robert D. Malmstrom, Rommie E. Amaro, Andrew D. McCulloch

**Affiliations:** ^1^Department of Bioengineering, University of CaliforniaSan Diego, La Jolla, CA, USA; ^2^Department of Chemistry and Biochemistry, University of CaliforniaSan Diego, La Jolla, CA, USA; ^3^National Biomedical Computation Resource, University of CaliforniaSan Diego, La Jolla, CA, USA; ^4^Department of Medicine, University of CaliforniaSan Diego, La Jolla, CA, USA

**Keywords:** protein kinase A, multiscale model, molecular dynamics, Brownian dynamics, Markov state model

## Abstract

The goal of multiscale modeling in biology is to use structurally based physico-chemical models to integrate across temporal and spatial scales of biology and thereby improve mechanistic understanding of, for example, how a single mutation can alter organism-scale phenotypes. This approach may also inform therapeutic strategies or identify candidate drug targets that might otherwise have been overlooked. However, in many cases, it remains unclear how best to synthesize information obtained from various scales and analysis approaches, such as atomistic molecular models, Markov state models (MSM), subcellular network models, and whole cell models. In this paper, we use protein kinase A (PKA) activation as a case study to explore how computational methods that model different physical scales can complement each other and integrate into an improved multiscale representation of the biological mechanisms. Using measured crystal structures, we show how molecular dynamics (MD) simulations coupled with atomic-scale MSMs can provide conformations for Brownian dynamics (BD) simulations to feed transitional states and kinetic parameters into protein-scale MSMs. We discuss how milestoning can give reaction probabilities and forward-rate constants of cAMP association events by seamlessly integrating MD and BD simulation scales. These rate constants coupled with MSMs provide a robust representation of the free energy landscape, enabling access to kinetic, and thermodynamic parameters unavailable from current experimental data. These approaches have helped to illuminate the cooperative nature of PKA activation in response to distinct cAMP binding events. Collectively, this approach exemplifies a general strategy for multiscale model development that is applicable to a wide range of biological problems.

## Introduction

**Figure 1:**
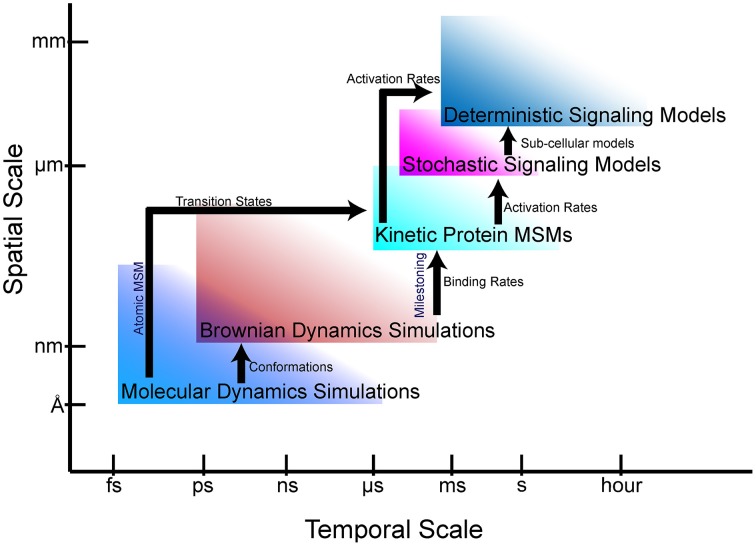
**Bridging gaps through multiscale modeling**. Simulation and modeling methods are limited in the spatial and temporal scales that can be represented. Arrows show the information that can be fed from one simulation regime to another.

The goal of multiscale modeling is to understand how the hierarchy of biological structures integrates to produce biochemical, cellular and physiological functions. At the single cell scale, signaling networks are analyzed using system analysis methods to provide mechanistic they are insights into the functional interactions between proteins and second messengers. Network models of cell signaling have recently been developed for neurons (Cowan et al., 2012), myocytes (Bondarenko, 2014), and pancreatic beta cells (Wang et al., 2012), to name a few. These cell-scale network models are helpful to understanding normal cell physiology, pathobiology, and therapeutic mechanisms. Interest in the phenomenological effects of protein mutations (Kirchner et al., 2012; Cong et al., 2013) are driving the development of new methods to incorporate atomic and molecular-scale models and data into whole cell simulations. To this end, advances in atomic-scale modeling, particularly molecular dynamics (MD) and Brownian dynamics (BD) simulations, have provided insights into the effects of mutations on protein folding and protein-protein interactions (Kozack and Subramaniam, 1993; Cregut and Serrano, 1999; De Rienzo et al., 2001; Koukos and Glykos, 2014). However, bridging these scales and disciplines to create models that can predict the effect of a point mutation or post-translational modification on cellular phenotypes remains a daunting task. Frequently, even nomenclature does not readily transcend disciplines, making interdisciplinary collaborations across scales more difficult. Furthermore, understanding the limitations of models and methods at each scale to avoid error propagation is essential to obtaining physiologically meaningful solutions. In this article, we describe atomic and protein-scale Markov state modeling (MSM), as well as milestoning, which allow us to bridge atomic-scale molecular models to cell-scale signaling networks (Figure 1).

Over the past decade, the availability of high-resolution protein structures and the capabilities of atomistic molecular modeling techniques has improved dramatically. MD and atomic-scale MSMs use atomic-resolution structural data to model the position of atoms in a protein and calculate the forces between them. This is helpful in predicting functional states and rates of conformational change. However, these methods cannot easily calculate the rates of interactions between molecules, which are needed for higher scale reaction network models.

Advances in BD simulations and milestoning have provided tools that are specialized in calculating diffusion-limited association rate constants. Previously, the data used for parameterization of the transitions in protein-scale MSM came almost exclusively from *in vitro* experiments where conditions are controlled to limit the number of potential states. These data included phosphorylation rates, k_on_/k_off_ of binding events, and ion channel transitions (Clancy and Rudy, 1999; Campbell et al., 2010; Boras et al., 2014). However, many molecular events occur at time-scales that cannot be easily accessed by experiments (Zhou and Bates, 2013). Fortunately, computational simulations have provided alternative methods for determining parameters for whole-cell models. BD simulations rely on simplifying assumptions that allow simulations of microscopic events that span larger systems and timescales than more detailed methods, such as MD, allow. BD can be used to determine association rate constants (k_on_) for diffusion-limited protein-protein and protein-small molecule interactions. It specifically examines how electrostatic and steric properties of molecules affect molecular encounter rates. Combining this information with *in vitro* experiments and MD-derived states will enable a new generation of protein-scale MSMs to be developed for incorporation into whole cell models.

As an example problem necessitating the integration of approaches across a broad range of spatial and temporal scales, we focus here on protein kinase A (PKA), which is activated by cAMP and is a key regulator of many cellular processes. In cardiac myocytes, for example, PKA is a critical regulator of intracellular calcium handling cycling, and its dysregulation is well known to be a contributing factor in heart failure (Bers, 2001). The PKA holoenzyme consists of two regulatory (R) subunits and two catalytic (C) subunits. Each R subunit has two cAMP-binding domains (CBD), a DD-docking domain, and a disordered linker region containing the inhibitory sequence that interacts with the C subunit. PKA is activated upon cAMP binding to the CBDs on the R subunit inducing release of the C subunit. Over the last 15 years, several whole-cell models of ventricular myocytes that incorporate calcium release and beta-adrenergic stimulation through a simplified PKA activation mechanism were developed (Saucerman et al., 2003; Bondarenko, 2014). More recently, a mechanistic protein-scale MSM of PKA holoenzyme activation was developed (Boras et al., 2014). Still, incorporating an improved PKA MSM into existing whole cell models will provide a more physiological testing of PKA activation as well as the capability to predict the effects of PKA mutations on the whole cell scale.

In this review, we highlight some of the tools and techniques used to develop integrative models that span scales from the molecule to the cell, including: MD, atomic MSM, BD, milestoning models, protein MSM, and whole cell modeling. We provide the nomenclature necessary to bridge these scales and discuss the limitations of these approaches as well as ways to minimize error propagation. Finally, we show the role of MD and BD simulations have played in the development of a protein scale MSM of PKA RIα and discuss the role this new protein-scale MSM of PKA will play in existing whole cell models of cardiac function and disease states.

## Nomenclature

This paper deals primarily with Markovian models, or models that are only dependent on the current state of the model and not the history of the states it has visited. Both MSM and milestoning models operate under a Markovian assumption. Also, for this paper we use “atomistic” or “atomic-scale” to describe any model that treats atoms explicitly. This generally includes MD, MSM, BD, and milestoning. These models stand in contrast to “protein-scale” Markov models and cell-systems models which primarily focus on protein and cell function and general protein-protein and small molecule-protein binding events. Even though atomistic MSM and protein MSM are both Markovian models, they serve distinct purposes.

## Accessing the conformational ensembles of proteins

A protein's function is governed by its conformational ensemble, which can be modulated though mutations and intermolecular interactions (Tsai et al., 1999; Henzler-Wildman and Kern, 2007; Boehr et al., 2009; Teilum et al., 2011; Marsh et al., 2012; Motlagh et al., 2014). Therefore, to build multiscale models starting at the atomic scale, one needs to elucidate the key conformational states of a protein and the dynamics of those states from atomistic data associated with those states. This can be achieved through exploration and characterization of the protein's conformational ensemble. In this section, we review computational methods for modeling the conformational ensembles of proteins important in cell signaling. We begin with an overview of molecular dynamics simulation methods and conclude with a discussion on the use of MSM to determine the conformational ensemble more efficiently.

### Molecular mechanics and molecular dynamics simulations

Atomistic models of conformational ensembles can be computationally generated from molecular mechanics simulations. These simulations require two components: a force field that describes how the atoms interact with each other and a method for exploring the conformational ensemble (Karplus and McCammon, 2002; Adcock and McCammon, 2006).

To simplify the complex quantum mechanical interactions between atoms, molecular mechanics simulations use empirical force fields to describe the interactions between atoms. These force fields are described in terms of classical mechanics (Wang et al., 2001; Ponder and Case, 2003; Adcock and McCammon, 2006). For example, each atom of a system is described as a charged particle in space. Bonding interactions between atoms are described as springs using Hooke's law. Nonbinding interactions between atoms are described as Columbic and van der Waals interactions. Commonly used force fields include CHARMM (Brooks et al., 2009), AMBER (Cornell et al., 1995), OPLS (Kaminski et al., 2001), and GROMOS (Oostenbrink et al., 2004). While a discussion on force field selection is beyond the scope of this review, it is important to understand the assumptions and performance bias of a force field used in any simulation (Guvench and Mackerell, 2008; Vitalini et al., 2015).

The motion of the atoms resulting from the force field determines the conformational ensemble of the system. The motions of these particles are generally simulated either with Monte Carlo techniques that randomly sample conformational space, or through MD simulations, where Langevin's or Newton's laws of motion are solved over time (Karplus and McCammon, 2002; Adcock and McCammon, 2006). While MD is more computationally expensive than MC, it retains the temporal relationship between conformations, which is advantageous when quantification of kinetic parameters is desired. Popular MD programs include AMBER (Pearlman et al., 1995), CHARMM (Brooks et al., 2009), GROMOS (Christen et al., 2005), and NAMD (Phillips et al., 2005).

Theoretically, MD simulations can sample the entire conformational ensemble of a system given infinite simulation time. While certain specialized supercomputers have been built to sample into the millisecond range (Shaw et al., 2010), with current commodity-level resources, MD simulations can only continuously sample a system for a few microseconds at most, which is insufficient to effectively sample most ensembles, including the CBD. However, with the increasing performance of supercomputers, GPU-accelerated MD simulations (Götz et al., 2012; Pierce et al., 2012; Salomon-Ferrer et al., 2013), and the use of highly distributed computing (Pande et al., 2003; Kohlhoff et al., 2014), multiple parallel MD simulations can achieve total non-continuous sampling time approaching the high-microsecond to low-millisecond range. MSMs can subsequently be used to stitch together the many short-timescale simulations into one cohesive framework that allows the extrapolation of longer-timescale data. This MSM framework was used for the CBD system discussed below.

### Atomic-scale Markov state models of a conformational ensemble

**Figure 2:**
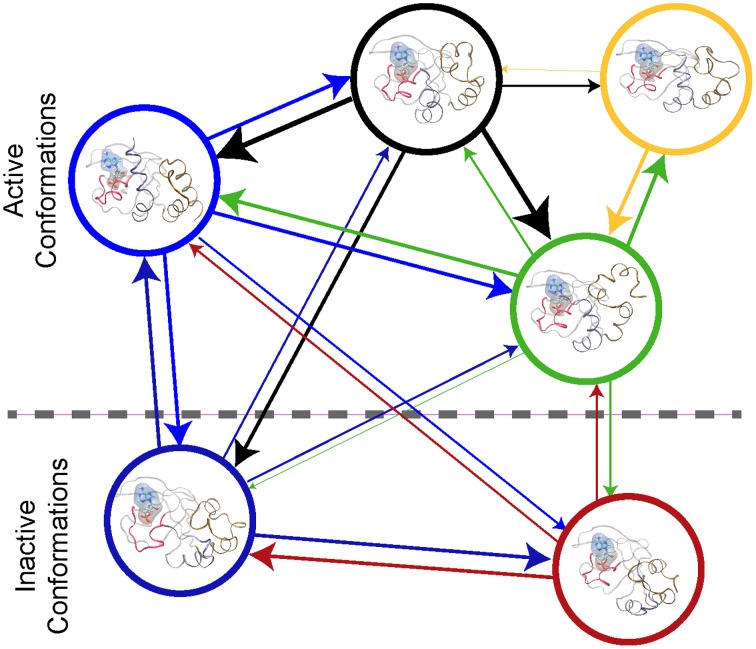
**Protein Kinase A cyclic nucleotide binding domain Markov state model**. This figure shows a graph repensantion the transtions between metastable states of the CBD with cAMP bound. Each node repesents the conformational state. The edges the transition between the node with their thickness being proportional to the probiblity of transtion.

An atomic-scale MSM describes the conformational ensemble of a protein as the probability of transitioning between discrete collections of conformational states at a fixed time (Pande et al., 2010; Chodera and Noé, 2014). This can be visualized as a bidirectional graph, (see Figure 2), where each node represents a cluster of similar conformations. The probability of transition between states is indicated by the thickness of the connecting lines in Figure 2. If the conformational states and the transitions can be accurately determined, then the MSM describes the thermodynamics and the kinetics of the system's conformational ensemble. Thus, one can derive the key parameters required for higher scale models with a MSM (Prinz et al., 2011a).

Atomic-scale MSMs of the conformational ensemble of a protein are built from MD simulations. Each conformation sampled during the simulation is assigned to a discrete conformational state, usually by clustering. Then the transitions between the discrete states are determined from the MD trajectory by counting the transitions. The transition counts are then used to generate a transition probability matrix, the mathematical representation of the MSM (Pande et al., 2010; Prinz et al., 2011a,b; Chodera and Noé, 2014). The transition probability matrix can be analyzed to determine the equilibrium population of each confrontational state, to identify metastable conformational states, to understand the principal motions of the protein, and to study the mechanisms of conformational will change (Pande et al., 2010; Prinz et al., 2011a,b; Chodera and Noé, 2014).

Because a MSM depends on the probabilities of transitions between discrete conformational states, the conformational ensemble of the protein can be sampled more efficiently than with traditional MD. To effectively sample the conformational ensemble of a protein at equilibrium using traditional MD simulations requires running the simulation long enough to explore the conformational ensemble multiple times. However, when building a MSM the MD simulations can be focused on the transitions between states avoiding spending time sampling stable conformations and improving the sampling of rare events. For example, a hypothetical transition between active and inactive states can be determined from multiple short simulations that explore the intervening conformations without requiring a single simulation to bridge the two states. Additionally, once a preliminary MSM is build poorly sampled transition can be additionally sampled to improve the quality of overall MSM.

Detailed methods for building MSMs for MD simulations have already been described (Sjoberg et al., 2010; Prinz et al., 2011b). Here we highlight key considerations for building a MSM that will be integrated with higher-scale models with examples from a recently developed MSM of the cyclic-nucleotide binding domain of the R subunit of PKA (Malmstrom et al., 2015). The overall process of building a MSM is as follows: (1) defining the conformational space; (2) initial molecular dynamics sampling; (3) iterative refinement of the MSM; and finally (4) selection of the final model for analysis.

The goal of our study was to determine the kinetics of the conformational ensemble of the CBD with and without cAMP bound. We defined the conformational space as the atomic coordinates of the alpha carbons in a protein, dividing the conformational states discrete into stats using RMSD-based clustering. We started sampling the CBD in either a crystallographic predetermined active or inactive state with and without cAMP bound. Building the final MSMs required over 70 μs of total sampling time comprised of both long-timescale initial sampling and iterative adaptive sampling to refine the models (Malmstrom et al., 2015).

Throughout the sampling and refinement process, the quality of a MSM is judged using implied timescale plots (Pande et al., 2010; Prinz et al., 2011b). Data points of the plot are constructed with eigenvalues of the transition probability matrix populated at different lag times, or times between events. The plots indicate at what lag times the models are Markovian and if the models are consistently capturing the principal conformational changes of the system. Additionally, a Chapman-Kolmogorov-test is used to validate the consistency of a MSM with molecular dynamics simulations (Prinz et al., 2011b). Using these two metrics, a final model is selected, the statistics of which are sampled at a specific lag time, which represents the fastest transition within the conformational ensemble that is also Markovian. This final model can then be used to derive the parameters for the multiscale model.

As described before, a MSM consists of the equilibrium probabilities for each conformational state. These probabilities are used to derive thermodynamic properties. Spectroscopic analysis can be used to identify metastable states within the conformational ensemble that can be used to build coarse-grained models of the system (Prinz et al., 2011a; Malmstrom et al., 2014). Transition path theory (Vanden-Eijnden and Tal, 2005) can be employed to approximate the kinetics of transitions between states. These rates become the parameters to feed into the multiscale model. For the CBD model we were able to obtain the rates of transitions between the active and inactive states and show how cAMP modulates the conformational ensemble, changing the function of the CBD. These rates have been an important benchmark in understanding the dynamics of the CBD, and form the foundation for examining the total R subunit and its interactions with the C subunit.

While the use of MSMs provides to conformational ensembles, there are still several important considerations and limitations to this method that should be considered in context of integrating them into a multiscale model. First, because conformational space is discretized, all kinetic rates are artificially fast (Prinz et al., 2011a,b), and should be considered an upper bound, especially when applied to high scale models. Second, a recent study indicates that modern force fields used in MD simulations produce varying transition kinetics (Vitalini et al., 2015). Therefore, the same force field should be used for all models of a system, and the limitations of the force fields should be understood. Thirdly, while the MSM is somewhat robust to errors in clustering, give a sufficiently fine division of conformational space (i.e., a lot of clusters) (Prinz et al., 2011b), the MSM is still dependent on the starting conformation used to initialize the simulations and the limitations of MD. Therefore, it is possible to not have included important conformational states leading to an incomplete model of the conformational landscape and incorrect predictions. However, limitations can be overcome using enhanced sampling methods (Bernardi et al., 2015) and from understanding acquired in the large-scale models. Finally, the MSMs are computationally demanding. This cost limits their usefulness in multiscale models, as a significant amount of time can be required to describe only one state in a higher scale model. Other sampling methods may be sufficient to obtain parameters for larger models. For example, if the opening and closing of a flap on a protein is the only permutation of interest, elastic network models are more computationally efficient in estimating those rates than MSM.

## Investigating intermolecular interactions

As we extend into larger spatial scales of modeling, the focus of our discussion shifts from intramolecular investigations with MD to the study of intermolecular encounters using BD. BD simulations are used to estimate the rate constants of second-order association events between two molecules. The output of these simulations provides kinetic on-rates used directly in higher levels of modeling. The application of BD simulations has extended beyond bi-molecular encounters in simulations of molecular crowding (McGuffee and Elcock, 2010) in cellular environments. In this section, we discuss the methodology and limitations of BD simulations, what can be gained from their use, and a brief overview of their application to multiscale modeling.

### Brownian dynamics simulations

In BD, molecular diffusion is modeled using the theory of Brownian motion; where internal dynamics of each molecule are frozen, constraining the molecules into rigid bodies that are free to diffuse and tumble in solution, but may not change shape. Popular programs used to carry out BD simulations include BrownDye (Huber and McCammon, 2010), SDA (Gabdoulline and Wade, 1997, 1998), ReaDDy (Geyer, 2011; Schöneberg and Noé, 2013), Brownmove (Geyer, 2011), and BD_BOX (Dlugosz et al., 2011). Similar to MD, one must choose a force field for BD simulations of the molecular system: AMBER (Dickson et al., 2014), CHARMM (Klauda et al., 2010), GROMOS (Oostenbrink et al., 2004), etc. However, the only force field quantities utilized in BD simulations are the partial charges and Van der Waals radii of each of the atoms of the biomolecule. In conjunction, these properties can be used to obtain the electrostatic potential from software that can solve the Poisson–Boltzmann (PB) equation. The electrostatic potentials of the biomolecules determine the long-range forces that the molecules impose on each other. Thus, electrostatics function as one of the most important determinants of the outcomes of BD simulations. Popular software packages that solve the PB equation include APBS (Baker et al., 2001; Holst, 2001) and DelPhi (Honig and Nicholls, 1995; Rocchia et al., 2002). Rigorous derivations and discussions of the form and proper usage of the PB equation can be found in the literature (Leach, 2001; Fogolari et al., 2002; Gu and Bourne, 2009).

In BD simulations, the solvent is modeled as a continuum; that is, there are no water molecules or dissolved ions modeled in atomic form in the simulation. Instead, the solvent is modeled as a field that surrounds the biomolecules and can have varying degrees of physical realism. This significantly reduces the computational power necessary for BD simulations in comparison to explicit solvent MD. The user typically specifies parameters that control solvent dielectric, hydrodynamics, desolvation, and ion screening, all which affect the realism of the solvent model and the computational cost of the simulation.

In addition to the long-range forces imposed by inter-molecular electrostatics, a stochastically determined force is also imposed on the molecules in a BD simulation. This stochastic force is directed randomly with a magnitude sampled from a Gaussian distribution centered at zero whose variance depends on the simulation time-step and the molecule's diffusivity properties. The stochastic force is intended to approximate the random “kicks” that would be caused by the solvent, but are otherwise absent in the continuum model.

Finally, the simulation must ensure that the Van der Waals radii of the atoms of different molecules do not overlap; a phenomenon known as a steric clash. Often, simulation steps that result in a steric clash are discarded and recomputed. Alternatively, many BD programs can impose a Lennard-Jones force at close molecular proximity to prevent a steric clash (Elcock, 2004; Huber and McCammon, 2010). BD simulation and the theory behind it compose a rich and expansive field, and many sources exist to allow the interested reader to improve his or her knowledge and technique (Ermak and Mccammon, 1978; Allen and Tildesley, 1987; Gabdoulline and Wade, 1996; Elcock et al., 2001; Gabdoulline and Wade, 2002; Madura et al., 2002; Elcock, 2004).

### Considerations for Brownian dynamics simulations

A key starting point for BD simulations is the selection of the encounter complex, which describes the atomic interactions that define a reaction between molecules. Ideally, crystal structures will inform this step. If crystal structures of the encounter complex do not exist, molecular docking programs can serve as a substitute. In the case of PKA, two crystallized structures of the regulatory subunit of protein kinase A RIα show very different conformations when bound to either cAMP (Su et al., 1995) or the catalytic subunit (Kim et al., 2007) To test the effects of structure on cAMP association with BD methods, one can use the crystal structure conformations of the regulatory subunit in separate BD simulations. Alternatively, the two different conformations can be used as starting points of separate MD simulations. A number of structures in the conformational ensemble will be generated and can serve as structures for separate BD simulations.

**Figure 3:**
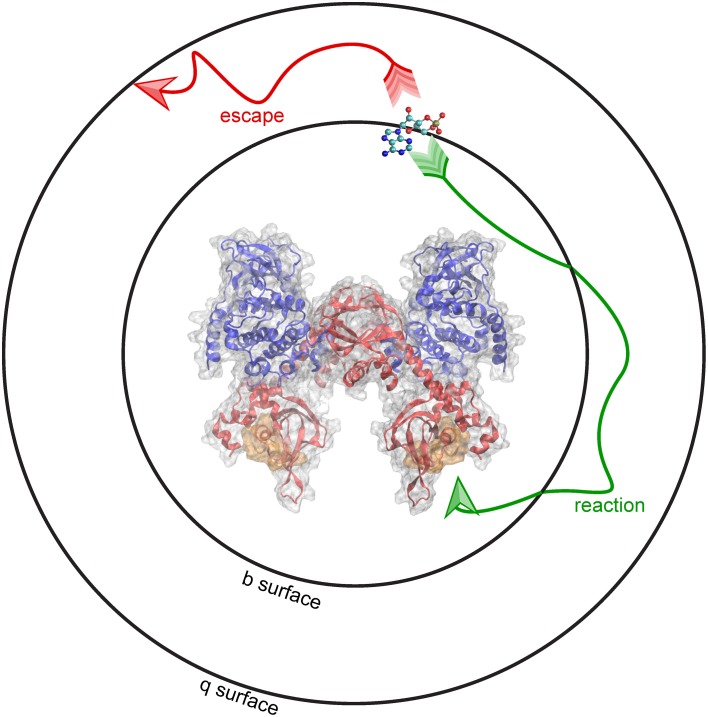
**Brownian dynamics simulation method**. BD simulations begin by placing molecules at a distance b from one another, shown here as a b-surface around PKA. When molecules diffuse toward the encounter complex (gold) a “reaction” (green arrow) occurs. Alternatively, molecules can “escape” (red arrow) by diffusing past a distance equal to q, shown here as the q-surface.

At the start of a simulation, the ligand is placed at a distance *b* from the receptor, at a location known as the *b surface*, which is defined as the distance where forces between the two molecules are centrosymmetric. Simulations terminate either upon the molecules reaching the predefined bimolecular encounter complex (a binding event), or when the molecules separate beyond a greater intermolecular distance *q*. The distance *q*, the radius of the *q surface*, is typically 10–50 nm larger than the distance *b* (Gabdoulline and Wade, 1998). The probability of association vs. escape is then used to calculate the association rate constant (k_on_). This schematic, including the surfaces at the *b* and *q* distances, are depicted using PKA as the receptor and cAMP as the ligand (Figure 3). BD can be used to model the association of cAMP with PKA, and predict features of the binding event, including the route of approach, the encounter complex, and the rate constant of association.

A second important factor in BD simulations is the structure of the molecules used in the simulations. Recall that BD simulations use a rigid-body approximation of molecules, meaning that the conformation of the molecule will not change throughout the simulation as it does in MD. Typically, crystal structure conformations are used. Another attractive possibility is the use of conformations generated by MD as starting points for BD simulations. Using this method, the user can select meta-stable or even rare conformations of a protein generated in MD simulations and compare the association rates and probabilities with respect to structural changes in the protein. MD trajectories can also be used to generate ensemble-averaged electrostatics (Votapka et al., 2013) where the simulated molecular motions are combined to form an electrostatic potential that includes the dynamic properties of the molecule. This effectively leads to a more holistic, dynamic representation of the electrostatic potential, effectively mediating some of the limitations of the rigid-body approximation of the simulations.

Solutions to the Poisson-Boltzmann equation include variations in the dipole moment and especially the charge density, with respect to how the solute affects the solvent, but also how the solvent affects *itself*. So while common implementations of the Poisson-Boltzmann equation solvers do not include many features of true aqueous solvents, it at least does assume that certain aspects of the solvent are heterogeneous. In addition, BD simulations themselves often model such things as hydrodynamics and desolvation forces, which are intended to approximate additional solvent features such as inertia and entropy at a surface, respectively.

Despite their ability to calculate association rate constants with respect to steric and electrostatic properties of molecules, BD simulations have limitations that users should know and recognize. First, the results of BD simulations depend on the encounter complex criteria. Such criteria must usually be tested and optimized in order to reproduce a reasonable association rate constant. Incorrectly chosen encounter criteria can significantly limit the accuracy of the simulation outcomes. Second, the rigid-body approximation of molecules in BD can only represent one part of the binding process: the diffusion—meaning that the rate constant calculated by simulations is that of association and not actual binding. Nevertheless, alternative methods that combine intermolecular investigations of BD with intramolecular dynamics of MD are being developed that promise kinetic rate estimations through simulation (Votapka and Amaro, 2015). These developments represent an approach toward spanning the MD and BD simulation regimes into a unified multiscale framework.

To our knowledge, no systematic method yet exists for estimating the true amount of error propagated by the assumptions inherent in BD. However, general consensus agrees that BD performs relatively well if the rate constants of an event it is estimating can be classified as a diffusion-limited process; that is, a process whose time to completion is primarily limited by particle diffusion. In the case of binding, the range of diffusion-limited rate constants is considered to include values of approximately 10^8^–10^9^ M^−1^s^−1^(Bar-Even et al., 2011).

Schemes do exist to approximate the precision of a k_on_ based on the statistical sampling of a binding probability vs. escape. Specifically, the uncertainty of the rate constant of binding is proportional to the inverse square root of the number of trajectories in BD simulations that have completed in a binding event. Since millions or even billions of BD trajectories can usually be completed at relatively little computational cost, it is typically not difficult to obtain relatively high precision of a rate constant using BD. However, while the estimated rate constant may be precise, it still may be inaccurate if the rigid molecules, implicit continuum solvent, or some other approximation assumed by BD do not adequately model the system. Comparison to experimental rate constants of the simulated ligand-receptor system, or perhaps of similar systems, can give an indication of the discrepancy between the “true” value, and the value obtained using BD.

### Unifying MD and BD simulations through milestoning

The possibility of combining the speed of rigid-body BD simulations with the flexibility of all-atom MD simulations to predict kinetic and thermodynamic quantities of interest is an attractive option. Ensembles of conformations or trajectories can be sampled from each simulation method, and statistics involving the probability and timescales of transitions between predefined states from the simulations can be combined using MSMs or the theory of milestoning (Faradjian and Elber, 2004) to model the details of intermolecular interactions.

Milestoning is a technique that is similar to the theory used in MSMs and can serve as an alternative approach to investigating biomolecular events, such as conformational sampling (West et al., 2007; Mugnai and Elber, 2015), diffusion (Mugnai and Elber, 2015), and membrane permeation (Cardenas et al., 2012), among others. Milestoning retrieves the kinetics as well as the thermodynamics of chemical processes (Vanden-Eijnden et al., 2008; Májek and Elber, 2010; Kirmizialtin and Elber, 2011), and can make use of extensive parallelization. Although similar to MSMs, milestoning models have a number of key differences, and may or may not be well suited to address a particular biophysical question. Unlike MSM states that are volumes in phase space where the system exists until it crosses into another, milestones are surfaces in phase space that the system traverses, and where the system's current “state” is the surface that the system has most recently crossed.

**Figure 4:**
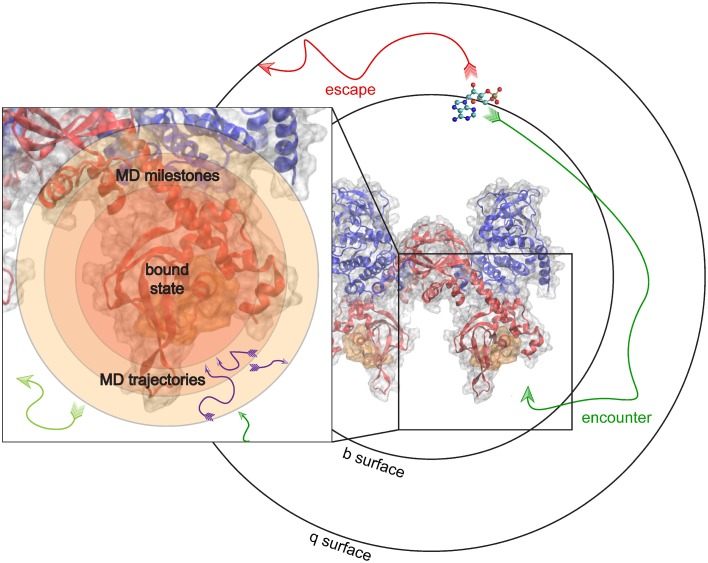
**Milestoning applied to unite MD and BD**. MD and BD Simulations are run to populate transition times and probilities in a milestoning model of cAMP binding to PKA. BD simulations are used to model an encounter event, and subsequent MD simulations model the details of the actual binding or reaction event.

To give an example, we examine the hypothetical case where the k_on_ of binding between PKA and cAMP can be predicted. In this milestoning model, we define a set of concentric spheres of different radii, all centered on the binding site of PKA (Figure 4). These concentric spheres define the milestones. MD simulations are started from conformations where cAMP is located on each spherical milestone, and each simulation is similarly terminated once cAMP diffuses to another surface. Thus, to the milestoning model, whichever simulation method is used to populate the transition kernels and incubation time vectors with statistics is of no consequence. The most appropriate simulation method can be chosen when cAMP is started on a particular surface.

Unlike MSMs, milestoning transitions may only occur to states that are adjacent in the positional or conformational space and lag times (or incubation times) can vary between inter-state transitions. Therefore, milestoning may be a desirable technique in situations where the system crossing surfaces would more appropriately represent transitions than the system traversing regions of space. For instance, because current implementations of BD simulations make extensive use of surfaces, such as the surfaces at the *b* and *q* distances and the encounter surfaces, milestoning is a natural choice to utilize transition statistics obtained in BD simulations. MD simulations modeling a binding event can make use of either milestoning models or MSMs, but when a combination with BD is desired, milestoning offers a promising framework to combine statistics from the two simulation methods.

Milestoning theory can be used to investigate a wide diversity of biophysical scenarios, and has been applied in a variety of contexts (Faradjian and Elber, 2004; Elber, 2007; West et al., 2007; Elber and West, 2010; Cardenas et al., 2012). In some physical situations, implementations of milestoning outperformed MSMs in resemblance to experimental results (Vanden-Eijnden et al., 2008). The application of milestoning to intermolecular interactions is still a recent development, and many possible improvements may enhance the efficiency and accuracy of the estimation of binding rate constants. Examples of these include further discretizing the system into grid-like milestones, rotational milestones, or milestones that can represent internal degrees of freedom. Extensive derivations and discussions of milestoning theory are discussed elsewhere (Faradjian and Elber, 2004; Vanden-Eijnden et al., 2008; Májek and Elber, 2010; Kirmizialtin and Elber, 2011).

## From atomistic to protein-scale models

Bridging the gap from atomic simulations to whole cell models is challenging. Protein-scale MSMs connect the atomistic scale to cell or tissue phenomena by reducing the complexity of molecular models. This enables simulations on larger time and spatial scales, while maintaining structural details required for protein function. These models simulate biological phenomena relevant to a whole-cell model, including ionic currents, fraction phosphorylated, or percent activation, and the output can be compared to *in vivo* experiments.

Protein-scale MSMs have been used to represent protein interactions since the early-1990's (Edeson et al., 1990). Several papers have been written on the development of protein-scale MSM, particularly of ion channels (Edeson et al., 1990; Giugliano, 2000; Gurkiewicz et al., 2011; Lampert and Korngreen, 2014). Ion channel MSMs have been made possible by the detailed statistical data that comes from single channel patch clamp recordings (Qin et al., 1996). These models have started to replace traditional phenomenological Hodgkin-Huxley style models of ion channel kinetics in whole cell action potential models (Rudy and Silva, 2006). They have been most useful when there is a need to model the effects of specific channel modifications, such as drug binding (Clancy et al., 2007), gene mutation (Rudy and Silva, 2006), or post-translational modifications (Yang and Saucerman, 2012). But the use of protein-scale MSMs is not limited to those systems where dynamic biophysical recordings are available; instead, these models can be built from BD and MD simulations.

### Protein-scale MSM

The first step in model development is to determine the overall structure of the model. Unlike atomic-scale MSMs, protein-scale MSMs do not represent every conformation of atoms as a state; instead, each state represents an ensemble of related atomic conformations that comprise a functional structure. This significantly limits the number of degrees of freedom and decreases the computational power needed, which enables multiple protein-scale MSMs to be combined into system-scale models. However, because these models are a simplification of the total potential states, the choice of which states are relevant becomes essential to making a useful model of a protein.

#### Functional state discovery through MD simulations

Frequently, several different states are captured by molecular-scale experiments, including X-ray crystallography and mass spectrometry. These experimental approaches can provide data on particular stable conformations (e.g., active or inactive states); however, due to the static nature of these tools, significantly less information is known about the transitions between states. For example, there are published structures of the R subunit of PKA bound either to cAMP or to the C subunit, but little is known about the transition between these end states. MD simulations can suggest intermediate states for incorporation into protein-scale MSMs. Similarly, atomic-scale MSMs provide insights into which states are populated and the rates of transitions between conformations.

#### Using BD simulation to inform kinetics

For small molecule-protein interactions and protein-protein interactions, BD and experimental data can serve complementary roles in determining kinetics. Dissociation-rates are typically slower than association rates and are therefore easier to measure experimentally using techniques such as surface plasmon resonance (Herberg et al., 1996). Additionally, most dissociation events are limited by conformational changes and not by diffusion—the latter of which BD is designed to model. For these same problems, MD simulations would be required to run for inaccessibly long periods of time (ms to s) to register release events. Association-rates, on the other hand, tend to be orders of magnitude faster and therefore are harder to measure experimentally. BD simulations are ideal for measuring fast interaction rates on the ns to μs time scales, many of which are limited by diffusion. In combination with equilibrium data, these techniques can be used synergistically to determine rates for small molecule-protein and protein-protein interactions. By basing the ensemble of states of the model on MD simulations, and the kinetic interactions on BD simulations, it is possible to predict the effect of a mutation on protein function and, by extension, on the whole cell.

#### Testing with empirical data

Data from experiments, MD simulations, and BD simulations can be integrated into a simplified protein-scale MSM with states and interactions relevant to protein function. Frequently, these combined methods will suggest several possible functional state ensembles. Competing models are generated, with different states or different relationships between the states. Subsequently, the resulting models are tested to determine their ability to fit relevant experimental data (Boras et al., 2014). For protein-scale MSMs, the data used for fitting most often comes from *in vitro* experiments. Ideally, the data used to differentiate between competing models is collected under conditions that are most relevant to a whole cell. For example, in the PKA-RIα model developed by Boras et al. (2014) all of the data used for fitting was collected in the presence of excess Mg^2+^ and ATP, both of which have been shown to affect PKA activation (Neitzel et al., 1991). These conditions are similar to what is found in a cell; however, recently published data has also highlighted the role of ADP in PKA activation (Khavrutskii et al., 2009), which could affect the role of PKA in metabolism but is absent in the current MSM.

The accuracy of each theoretical model is determined using an error function based on the weighted sum of squares difference between the model's predictions and the available experimental data. Minimizing this error function optimizes unknown parameters. If the MSM are nested (all possible states in a model with fewer degrees of freedom can be represented in the model with more degrees of freedom) then a statistical *F*-test can be performed to determine if the added degrees of freedom significantly improve the fit (Anderson and Conder, 2011). This ensures that MSMs do not become needlessly complex without an improvement in the accuracy of the model's predictions.

Frequently, data acquired with mutant proteins that cannot reach specific states is used to differentiate competing models. The MSMs are altered slightly by removing those states, without refitting any parameters, and the output is compared against the experimental results (Boras et al., 2014). For example Clancy et al. used MSM of a cardiac sodium channel to show that a mutation in its C terminus can lead to long-QT syndrome, which causes life-threatening arrhythmias (Clancy and Rudy, 2002). This highlights how protein-scale MSM based on atomistic data can predict the effect a mutation will have on the whole cell and eventually on the organ scale as well.

To mitigate error propagation when the protein-scale MSM is added to whole cell models, a sensitivity analysis can be performed to test the robustness of the solution (Campbell et al., 2010). In this process each rate is perturbed to determine its effect on the desired output of the model. States can also be removed to see how essential they are to the final result. The objective is to quantify how much the final result relies on any individual rate or state and compare that to the uncertainty in the experimental measurements. This technique can also highlight which states predicted from atomic scale modeling would have the greatest effect if mutated or pharmaceutically targeted. This is especially useful in quantifying the potential effect of rare conformations. Due to sampling bias they may not be captured in MD simulations but by adding them to the model their potential effect can be determined even if precise kinetic parameters are not known.

### Applying to MD and BD modeling to protein scale PKA-RIα MSM

**Figure 5:**
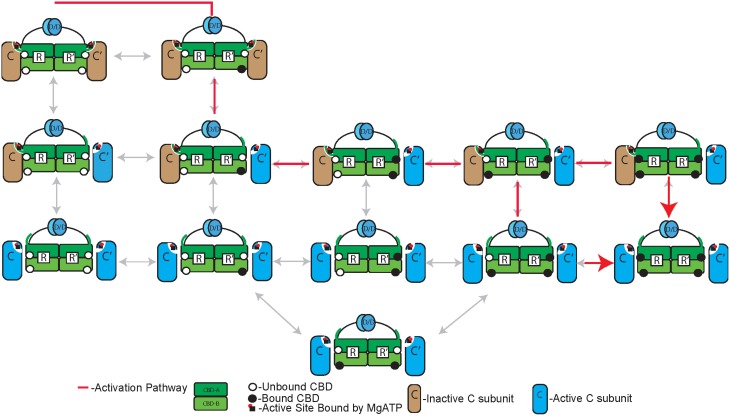
**The Markov State Model of PKA-RIα R_2_C_2_ holoenzyme**. A representation of MSM states for the activation of PKA-RIα R_2_C_2_ holoenzyme by cAMP first published in JBC (Boras et al., 2014). The red arrows represent the dominant pathway during activation. The two R- and C-subunits are identical but for simplicity of naming the first R-subunit to bind C-subunit is named R, while the first R-subunit to bind cAMP is R′.

These techniques have been applied to the development of a novel PKA protein scale MSM (Boras et al., 2014) (Figure 5). First, the effects of cAMP binding on CBD-A of the regulatory subunit of PKA were examined (Malmstrom et al., 2015). Using extensive all atom molecular dynamics simulations integrated with atomic-MSM, the conformation of the CBD with and without cAMP bound was determined. Conformational selection was identified as the general mechanism of allostery within a single CBD, which transitions between an active and an inactive conformation whether or not cAMP is bound. cAMP was found to regulate the function of the CBD by deepening the free energy landscape and selecting conformational states that favor the active conformation. Interestingly, cAMP modifies the transition rate between the active and inactive conformation and not the transition between the inactive and active conformations. Additionally, the roles of each of the signaling motifs in the CBD were elucidated.

Based on these findings and crystal structure data, five nested protein scale MSM were considered. Each model was structured to test competing theories of PKA R_2_C_2_ activation based on MD simulations. The crystal structures suggested that a model that treated each R-C heterodimer as independent would be insufficient to fit the data, due to the compactness of the R_2_C_2_ holoenzyme. Atomistic MSM predicted that a conformational selection mechanism would most accurately fit the data for isolated CBDs. The models were developed in the Virtual Cell computational environment (Moraru et al., 2008) before being translated into MATLAB to take advantage of optimization programs (Marsden et al., 2008). The models were fitted to kinase activity and cAMP binding data under physiological conditions (Christensen et al., 2003). Additionally, the various models were then compared to mutant PKA experimental data with either an inhibited CBD-A or CBD-B binding site. One model was shown to fit the wild type and predict the experimental results better than any other. This model validated the atomistic MSM by showing that CBD-B binding leads to release of the C-subunit prior to CBD-A binding similar to a conformational selection mechanism and created a thermodynamic protein-scale MSM of PKA activation.

However, since the fitting data, as well as the mutant data, were collected at long enough intervals that an assumption of thermodynamic equilibrium was valid, the resulting MSM could only reproduce equilibrium behavior. From a cellular perspective, PKA's response to a stimulus over time is essential to understanding PKA function. The single turnover rate in response to a stimulus has been implicated in activation due to A kinase anchoring proteins that bind PKA near one target (Scott and Santana, 2010). Therefore, the addition of kinetic rates would significantly increase the utility of the MSM a whole cell model.

Owing to the fast rate of activation of PKA in the presence of cAMP, the amount of experimental kinetic data on PKA activation, particularly on-rates, is limited. This is a problem ideally suited to solving using atomic simulations. The atomic scale MSM suggested that cAMP binding only affects the rate of transition from active to inactive states but not the reverse. BD simulations can be combined with experimental data to suggest binding and release rates for R-C and R-cAMP interactions. In conjunction with *in vitro* experimental data this type of data will allow the thermodynamic MSM to become a kinetic MSM better suited to whole cell scale analysis of signaling network properties.

## Integrating protein scale MSM into whole cell models

The potential of molecular and protein-scale models culminate in whole-cell and tissue-scale models that can predict phenotypes and mechanistically explain disease states. These models combine several MSMs to predict cellular responses to either internal or external stimuli by tracing behavior down to molecular interactions. When developing these protein-scale MSMs, it is best to keep in mind what broader biological function will be modeled at a larger scale since this will determine not only what states are relevant but also what type of model is best for a given phenomena.

### Stochastic and deterministic MSM in the whole cell

Some cell functions are best simulated using a continuum of species concentrations and smooth probability distributions, while statistically rare events are better modeled when individual molecules are tracked and the stochasticity of interactions is accounted for. Correspondingly, protein-scale MSM can be either stochastic or deterministic in nature. The stochastic models, like the atomic-scale MSM described earlier, are based on Monte Carlo simulations where the probability of transitioning between states is dependent on the kinetics of the binding and/or the conformational shift that each transition represents. This is the most accurate representation, where each event is dependent on the chance that two molecules will interact or that a conformation will be sampled based on random motion.

Many biological processes, such as calcium sparks in cardiac myocytes, can be explained with stochastic simulations. Calcium sparks occur when calcium is released from the sarcoplasmic reticulum via an isolated cluster of ryanodine receptor calcium release channels in the absence of a depolarizing event. In other words, a single cleft or a cluster of clefts acts differently than the rest of the cell. Whole-cell deterministic models require that every channel of a given type are identical and therefore every channel could be fractionally open but no one channel could be fully open while the others were fully closed without changing the conditions. Therefore, to model phenomena like this, a stochastic model is necessary. When translating these models up to the whole cell, the stochastic models are ideal for agent based spatial modeling tools, such as MCell (Kerr et al., 2008), where each molecule is tracked and diffusion is represented by a random walk; although, it is worth noting that a whole-cell model can consist of a continuum diffusion approximation but still contain stochastic protein-scale MSMs. Agent-based models are ideal for small numbers of molecules or short time and spatial scales, where tracking each molecule is computationally reasonable or average approximations may be invalid.

Over a long enough time-scale or a large enough population of molecules, the Monte Carlo simulation will approach the deterministic solution. The deterministic solution is represented by a system of ordinary differential equations, instead of being represented by a transition matrix of probabilities. In these models, the states of the MSM are frequently populated by concentrations instead of a specific number of molecules.

Many biological processes can be represented deterministically, most often when the system has a large number of molecules, or covers a long time and spatial scales. Models of the calcium concentration in a cell, for example, would require a deterministic model because computationally there are too many molecules to follow and the simulation becomes intractable. However, even on a small scale a deterministic approximation can be valid. For example, Hake et al. (2014) showed that for a single dyadic cleft in a cardiac myocyte, the random walk and the deterministic continuum approximation gives the same result for a calcium induced calcium release event, even though a continuum approximation of the calcium in the cleft is unrealistic due to the scarcity of calcium ions. By treating the continuum as deterministic but the protein-scale MSMs as stochastic we can reproduce the stochastic sparks while limiting the required computational power.

### Advantages in whole cell modeling

The potential of molecular and protein-scale models culminate in whole-cell and tissue-scale models that can predict phenotypes and mechanistically explain disease states. These models combine several MSMs to predict cellular responses to either internal or external stimuli by tracing behavior down to molecular interactions. The power of building atomic-scale and protein-scale MSMs for wild type and disease mutants comes from their integration into whole cell models. At the whole cell scale, differences in sub-cellular dynamics of protein mutations can be studied comparatively with their wild-type counterparts. Several disease states come from known protein mutations. For example, in the case of PKA-RIα, 117 polymorphisms and mutations have already been discovered (Horvath et al., 2010). Owing to the complexity of signaling pathways, how these mutations affect cell function is frequently unclear but by creating a whole cell model from molecular mechanisms it is possible to predict how a given mutation will lead to a particular a cellular phenotype.

Whole cell models based on atomic resolution information have opened entirely new avenues of research into drug discovery. In addition to suggesting which protein is a viable target, mechanistic whole cell models can suggest which protein conformation is most favorable and even the chemical shape of a small molecule necessary to inhibit/promote activation. This allows a scale of specificity that could decrease toxicity and limit side effects.

Cardiac arrhythmias are a prime example of the potential relevance of whole cell models. Currently, one of the most commonly prescribed classes of drugs to treat arrhythmias are beta-blockers. Beta blockers bind the beta adrenergic receptors to inhibit epinephrine and norepinephrine binding to reduce the chance of a second heart attack (Cruickshank, 2010). However, this inhibits the entire beta-adrenergic pathway. By combining this new PKA protein-scale MSM with previously published adrenergic signaling models of the heart (Saucerman et al., 2003; Bondarenko, 2014), it is possible to suggest drug targets and even specific binding pockets to inhibit parts of the pathway while limiting their effect on the rest of the cell.

## Conclusions

For years, atomic-resolution protein structures have aided our understanding of protein function not only through static structures provided by NMR and crystallographic experiments, but also through the prediction of dynamic properties with MD simulations. MD has revealed ensembles of structures that comprise the conformational landscape of a protein. Due to computational limitations, classical MD simulations are only able to generate microseconds (or less) of simulated time. This significantly limits the extent of the protein conformational ensemble sampled. However, information generated by MD simulations can be integrated into atomic scale MSMs, which are used to link states generated in a conformational ensemble through a kinetic scheme. The outputs of structures from MD simulations are analyzed according to a chosen conformational state description and are discretized into microstates. The MD trajectory informs which transitory states are most favorable and calculates the transition rates between these states to be used in different scales of modeling.

MD simulations subsequently inform both the atomic scale MSMs and BD simulations. BD simulations typically use rigid-body representations; therefore selected conformations are important for understanding the effect of different structures on association probability. MD simulations provide relevant conformations for BD by generating stable conformations. In addition, ensemble-averaged electrostatics can be generated from the MD trajectories, reflecting the dynamic properties of a molecule in a static electrostatic potential map. Finally, MD and BD simulations can be directly integrated through milestoning to derive association rate constants (k_on_) of diffusion-limited processes; a process that combines the two distinct simulation regimes—utilizing the advantages and minimizing the disadvantages of each. Such a scheme can vastly expand the time and length scales accessible in the simulation of multimolecular interactions between proteins and small molecules and/or other proteins to be combined with experimental data in protein-scale MSMs.

Protein-scale MSMs draw on every facet of the atomistic models to bridge the atomic and cellular scales. MD simulations and atomistic-scale MSMs suggest which ensemble of states will reproduce a molecular function. BD simulations combined with milestoning predict association rate constants that would be difficult to experimentally reproduce. This information, when combined with *in vitro* experimental data and statistical analysis tools, leads to the development of protein-scale MSM's for incorporation into whole cell models. Whole cell models based on atomic level details provide a new scale of specificity. The ability to scale up the effects of a protein mutation on a cellular level function is the ideal goal of a robust MSM of this kind.

As discussed throughout this paper, during the process of multiscale modeling it is essential to consider error propagation, or the effect of inaccuracies in small-scale models and the translation of this error into higher levels. For example, conditions such as molecular crowding in the cell likely affect the energetic landscape of a protein, a phenomenon not explicitly represented in an MD simulation. The limited sampling time of MD simulations can bias the conformational landscape of the protein, affecting the kinetic rates determined by the MSM. Structures and kinetics abstracted from biased simulations can further limit the accuracy of BD simulations and protein-scale MSM, respectively. Furthermore, coarser-grained simulations such as BD and protein-scale MSMs are not free of their own inaccuracies. The sources of errors in many modeling methods may or may not be easy to recognized and the best practices for quantifying the errors is still an active area of research. Iterating through the multiscale modeling process is extremely time consuming, since frequently MSMs must be fully recreated when new constrains are added. With ample computational resources, a multiscale modeler can incorporate recursive feedback loops from multiple scales to converge to a steady solution of the represented system.

Building a multiscale model, despite inaccuracies, is extremely useful. The findings of larger scale models can be used to inform the finer scales, like the identification of unknown conformational states in protein-small molecule energetic landscape. Carefully considering the whole-cell constraints of a given disease state or drug target before creating the initial model can allow these multiscale models to be a powerful and efficient tool for understanding the mechanisms behind some of the most intriguing biological questions.

## Funding

This work was funded in part by the National Biomedical Computation Resource, NIH P41 GM103426, NIH Director's New Innovator Award Program DP2-OD007237 (to REA), through NSF XSEDE Supercomputer resources grant RAC CHE060073N (to REA), through NIH grants 5 P01 HL098053, 1 R01 HL105242, and 1 R03 EB014593 (to ADM) and by NSF graduate fellowship DGE-1144086 (to LWV).

### Conflict of interest statement

Andrew D. McCulloch is required to disclose that he is a co-founder and equity holder of Insilicomed, Inc., a licensee of software developed by his laboratory. This relationship has been disclosed to and is managed by the University of California, San Diego. The software was not used in this research, and the company has had no involvement in this research or any interest in its outcome. The other authors declare that the research was conducted in the absence of any commercial or financial relationships that could be construed as a potential conflict of interest.

## Abbreviations

PKA: Protein Kinase A
MD: Molecular Dynamics
MSM: Markov state model
BD: Brownian Dynamics.
